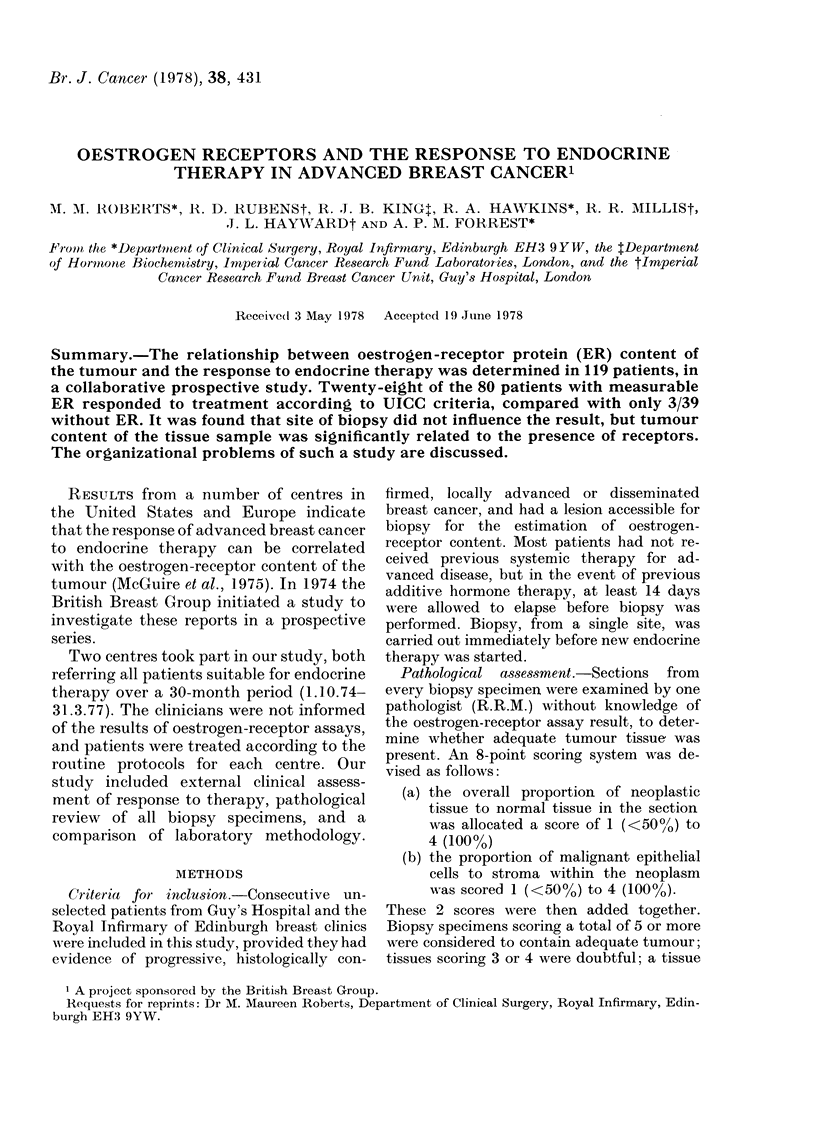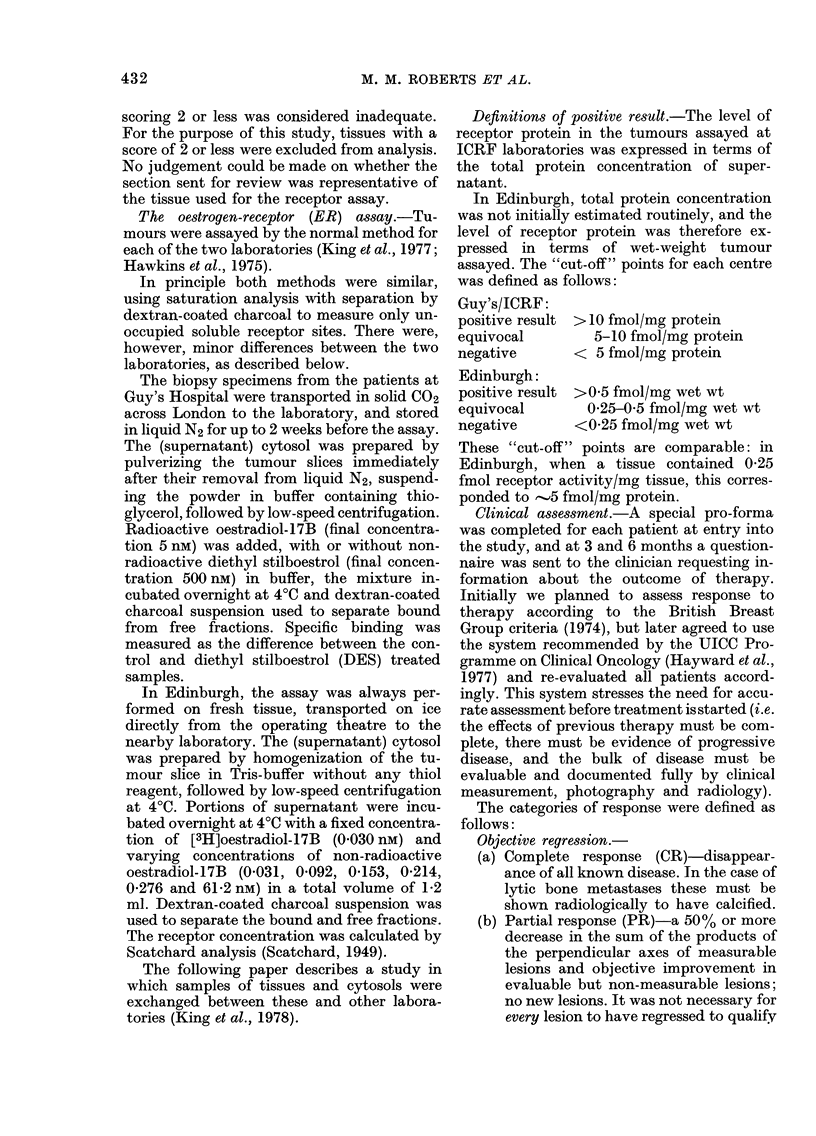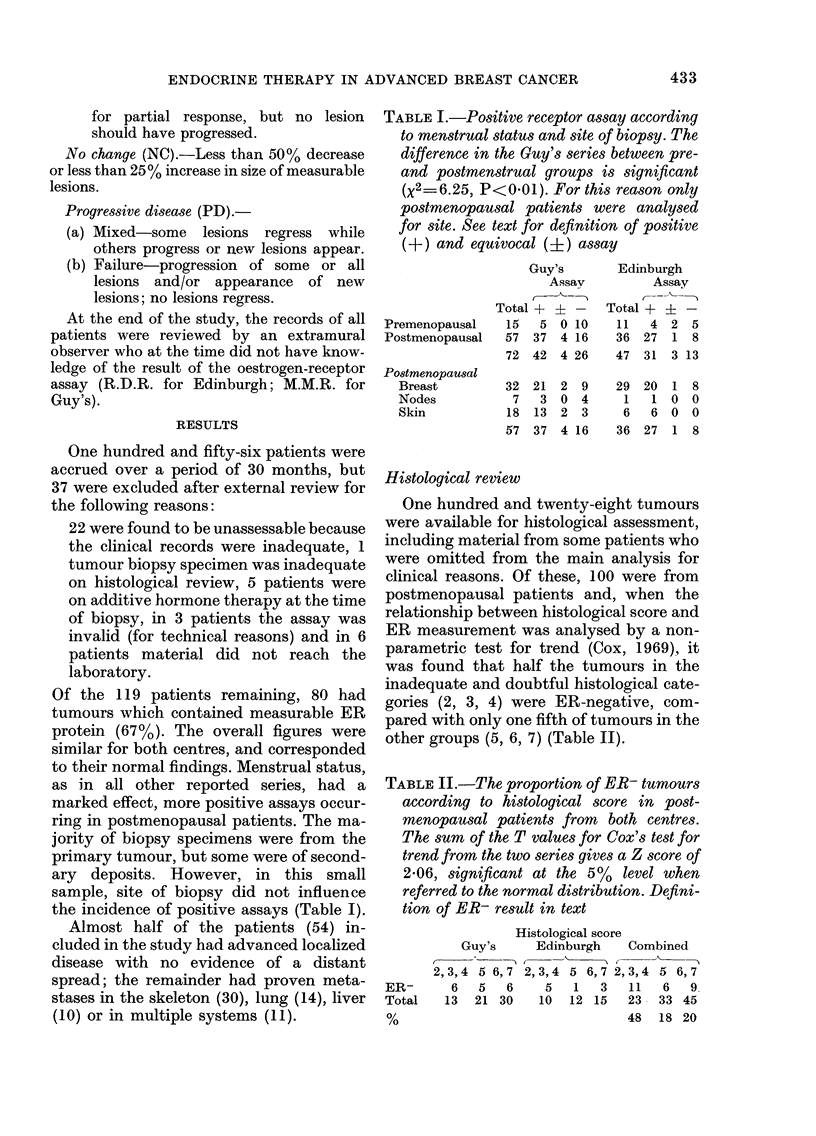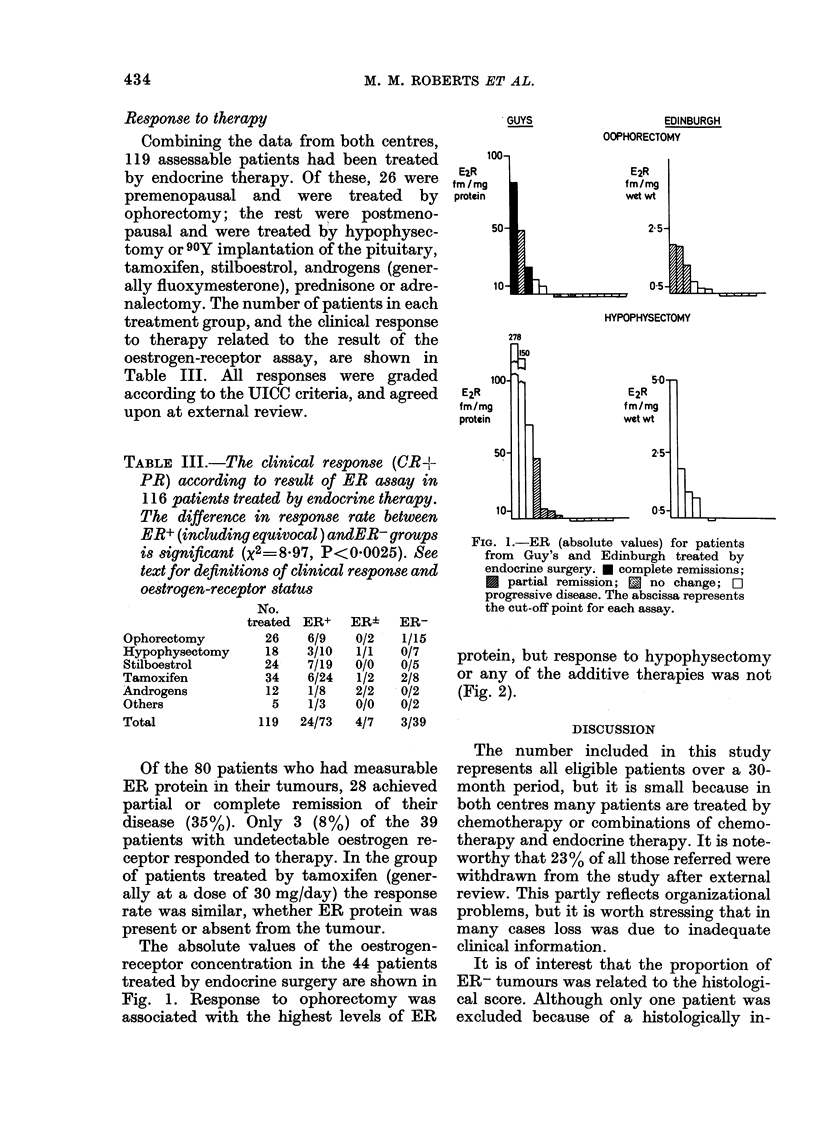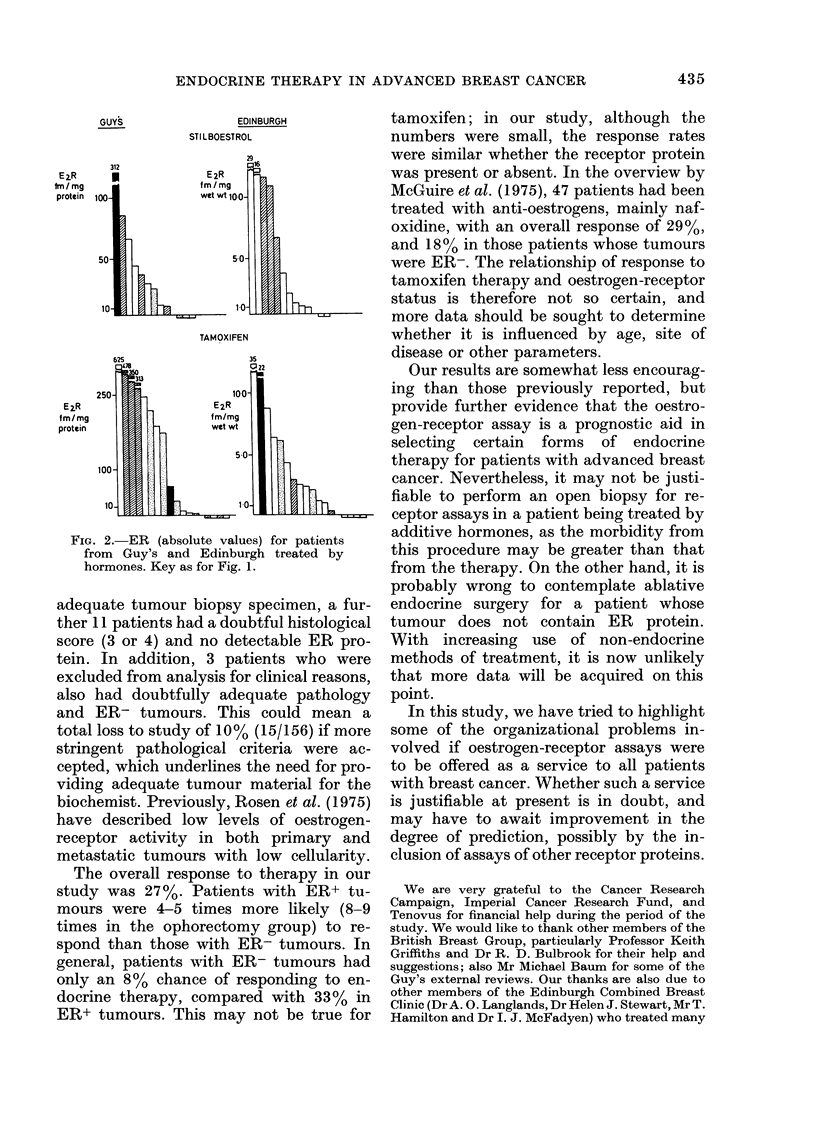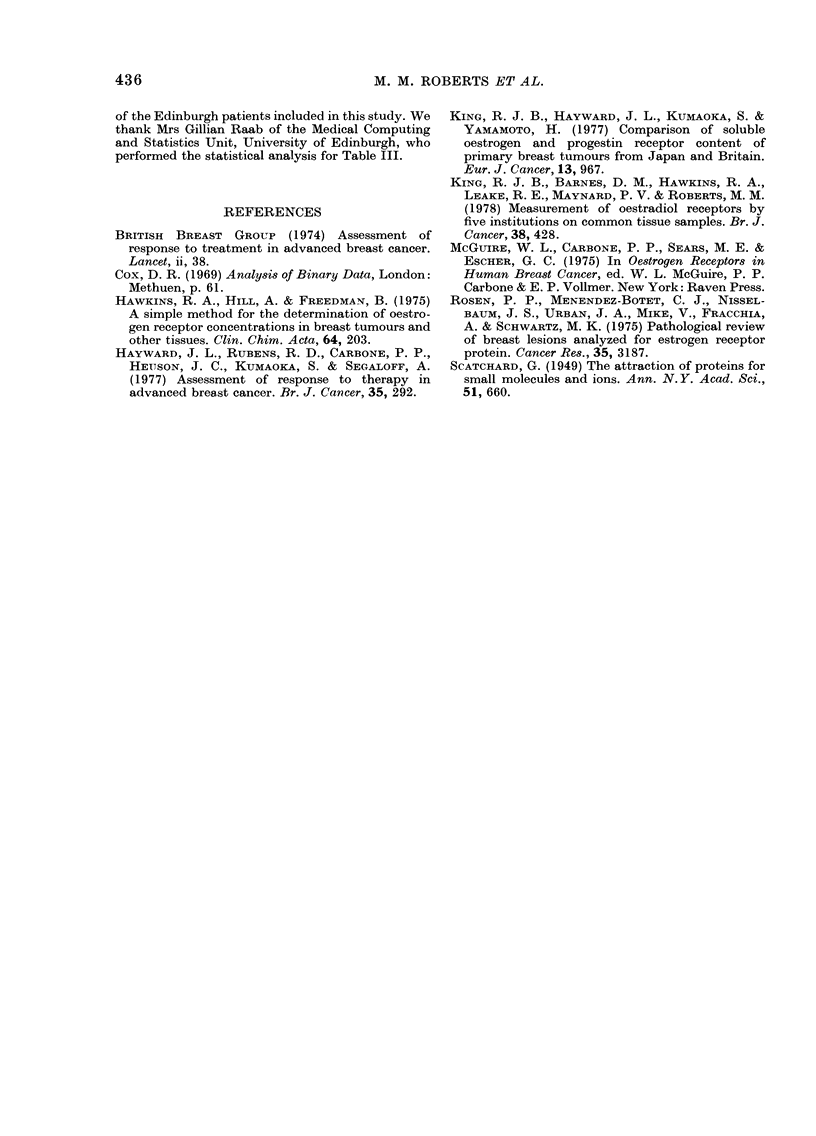# Oestrogen receptors and the response to endocrine therapy in advanced breast cancer.

**DOI:** 10.1038/bjc.1978.225

**Published:** 1978-09

**Authors:** M. M. Roberts, R. D. Rubens, R. J. King, R. A. Hawkins, R. R. Millis, J. L. Hayward, A. P. Forrest

## Abstract

The relationship between oestrogen-receptor protein (ER) content of the tumour and the response to endocrine therapy was determined in 119 patients, in a collaborative prospective study. Twenty-eight of the 80 patients with measurable ER responded to treatment according to UICC criteria, compared with only 3/39 without ER. It was found that site of biopsy did not influence the result, but tumour content of the tissue sample was significantly related to the presence of receptors. The organizational problems of such a study are discussed.


*Br. J. Cancer* (1978), **38**, 431

### OESTROGEN RECEPTORS AND THE RESPONSE TO ENDOCRINE THERAPY IN ADVANCED BREAST CANCER[1]

M. M. ROBERTS*, R. D. RUBENS†, R. J. B. KING‡, R. A. HAWKINS*, R. R. MILLIS†, J. L. HAYWARD† AND A. P. M. FORREST*

*From the *Department of Clinical Surgery, Royal Infirmary, Edinburgh EH3 9YW, the ‡Department of Hormone Biochemistry, Imperial Cancer Research Fund Laboratories, London, and the †Imperial Cancer Research Fund Breast Cancer Unit, Guy's Hospital, London*

Received 3 May 1978 Accepted 19 June 1978

**Summary.—The relationship between oestrogen-receptor protein (ER) content of the tumour and the response to endocrine therapy was determined in 119 patients, in a collaborative prospective study. Twenty-eight of the 80 patients with measurable ER responded to treatment according to UICC criteria, compared with only 3/39 without ER. It was found that site of biopsy did not influence the result, but tumour content of the tissue sample was significantly related to the presence of receptors. The organizational problems of such a study are discussed.**

RESULTS from a number of centres in the United States and Europe indicate that the response of advanced breast cancer to endocrine therapy can be correlated with the oestrogen-receptor content of the tumour (McGuire *et al.*, 1975). In 1974 the British Breast Group initiated a study to investigate these reports in a prospective series.

Two centres took part in our study, both referring all patients suitable for endocrine therapy over a 30-month period (1.10.74–31.3.77). The clinicians were not informed of the results of oestrogen-receptor assays, and patients were treated according to the routine protocols for each centre. Our study included external clinical assessment of response to therapy, pathological review of all biopsy specimens, and a comparison of laboratory methodology.

### METHODS

*Criteria for inclusion.*—Consecutive unselected patients from Guy's Hospital and the Royal Infirmary of Edinburgh breast clinics were included in this study, provided they had evidence of progressive, histologically confirmed, locally advanced or disseminated breast cancer, and had a lesion accessible for biopsy for the estimation of oestrogen-receptor content. Most patients had not received previous systemic therapy for advanced disease, but in the event of previous additive hormone therapy, at least 14 days were allowed to elapse before biopsy was performed. Biopsy, from a single site, was carried out immediately before new endocrine therapy was started.

*Pathological assessment.*—Sections from every biopsy specimen were examined by one pathologist (R.R.M.) without knowledge of the oestrogen-receptor assay result, to determine whether adequate tumour tissue was present. An 8-point scoring system was devised as follows:

(a) the overall proportion of neoplastic tissue to normal tissue in the section was allocated a score of 1 ($<50\%$) to 4 (100%)
(b) the proportion of malignant epithelial cells to stroma within the neoplasm was scored 1 ($<50\%$) to 4 (100%).

These 2 scores were then added together. Biopsy specimens scoring a total of 5 or more were considered to contain adequate tumour; tissues scoring 3 or 4 were doubtful; a tissue

[1] A project sponsored by the British Breast Group.
Requests for reprints: Dr M. Maureen Roberts, Department of Clinical Surgery, Royal Infirmary, Edinburgh EH3 9YW.

scoring 2 or less was considered inadequate. For the purpose of this study, tissues with a score of 2 or less were excluded from analysis. No judgement could be made on whether the section sent for review was representative of the tissue used for the receptor assay.

*The oestrogen-receptor* (*ER*) *assay.*—Tumours were assayed by the normal method for each of the two laboratories (King *et al.*, 1977; Hawkins *et al.*, 1975).

In principle both methods were similar, using saturation analysis with separation by dextran-coated charcoal to measure only unoccupied soluble receptor sites. There were, however, minor differences between the two laboratories, as described below.

The biopsy specimens from the patients at Guy's Hospital were transported in solid $CO_2$ across London to the laboratory, and stored in liquid $N_2$ for up to 2 weeks before the assay. The (supernatant) cytosol was prepared by pulverizing the tumour slices immediately after their removal from liquid $N_2$, suspending the powder in buffer containing thioglycerol, followed by low-speed centrifugation. Radioactive oestradiol-17B (final concentration 5 nM) was added, with or without non-radioactive diethyl stilboestrol (final concentration 500 nM) in buffer, the mixture incubated overnight at 4°C and dextran-coated charcoal suspension used to separate bound from free fractions. Specific binding was measured as the difference between the control and diethyl stilboestrol (DES) treated samples.

In Edinburgh, the assay was always performed on fresh tissue, transported on ice directly from the operating theatre to the nearby laboratory. The (supernatant) cytosol was prepared by homogenization of the tumour slice in Tris-buffer without any thiol reagent, followed by low-speed centrifugation at 4°C. Portions of supernatant were incubated overnight at 4°C with a fixed concentration of [$^3$H]oestradiol-17B (0·030 nM) and varying concentrations of non-radioactive oestradiol-17B (0·031, 0·092, 0·153, 0·214, 0·276 and 61·2 nM) in a total volume of 1·2 ml. Dextran-coated charcoal suspension was used to separate the bound and free fractions. The receptor concentration was calculated by Scatchard analysis (Scatchard, 1949).

The following paper describes a study in which samples of tissues and cytosols were exchanged between these and other laboratories (King *et al.*, 1978).

*Definitions of positive result.*—The level of receptor protein in the tumours assayed at ICRF laboratories was expressed in terms of the total protein concentration of supernatant.

In Edinburgh, total protein concentration was not initially estimated routinely, and the level of receptor protein was therefore expressed in terms of wet-weight tumour assayed. The "cut-off" points for each centre was defined as follows:

| | |
|---|---|
| Guy's/ICRF: | |
| positive result | $>$10 fmol/mg protein |
| equivocal | 5–10 fmol/mg protein |
| negative | $<$ 5 fmol/mg protein |
| Edinburgh: | |
| positive result | $>$0·5 fmol/mg wet wt |
| equivocal | 0·25–0·5 fmol/mg wet wt |
| negative | $<$0·25 fmol/mg wet wt |

These "cut-off" points are comparable: in Edinburgh, when a tissue contained 0·25 fmol receptor activity/mg tissue, this corresponded to $\sim$5 fmol/mg protein.

*Clinical assessment.*—A special pro-forma was completed for each patient at entry into the study, and at 3 and 6 months a questionnaire was sent to the clinician requesting information about the outcome of therapy. Initially we planned to assess response to therapy according to the British Breast Group criteria (1974), but later agreed to use the system recommended by the UICC Programme on Clinical Oncology (Hayward *et al.*, 1977) and re-evaluated all patients accordingly. This system stresses the need for accurate assessment before treatment is started (*i.e.* the effects of previous therapy must be complete, there must be evidence of progressive disease, and the bulk of disease must be evaluable and documented fully by clinical measurement, photography and radiology).

The categories of response were defined as follows:

*Objective regression.*—

(a) Complete response (CR)—disappearance of all known disease. In the case of lytic bone metastases these must be shown radiologically to have calcified.

(b) Partial response (PR)—a 50% or more decrease in the sum of the products of the perpendicular axes of measurable lesions and objective improvement in evaluable but non-measurable lesions; no new lesions. It was not necessary for *every* lesion to have regressed to qualify for partial response, but no lesion should have progressed.

*No change* (NC).—Less than 50% decrease or less than 25% increase in size of measurable lesions.

*Progressive disease* (PD).—

(a) Mixed—some lesions regress while others progress or new lesions appear.

(b) Failure—progression of some or all lesions and/or appearance of new lesions; no lesions regress.

At the end of the study, the records of all patients were reviewed by an extramural observer who at the time did not have knowledge of the result of the oestrogen-receptor assay (R.D.R. for Edinburgh; M.M.R. for Guy's).

RESULTS

One hundred and fifty-six patients were accrued over a period of 30 months, but 37 were excluded after external review for the following reasons:

22 were found to be unassessable because the clinical records were inadequate, 1 tumour biopsy specimen was inadequate on histological review, 5 patients were on additive hormone therapy at the time of biopsy, in 3 patients the assay was invalid (for technical reasons) and in 6 patients material did not reach the laboratory.

Of the 119 patients remaining, 80 had tumours which contained measurable ER protein (67%). The overall figures were similar for both centres, and corresponded to their normal findings. Menstrual status, as in all other reported series, had a marked effect, more positive assays occurring in postmenopausal patients. The majority of biopsy specimens were from the primary tumour, but some were of secondary deposits. However, in this small sample, site of biopsy did not influence the incidence of positive assays (Table I).

Almost half of the patients (54) included in the study had advanced localized disease with no evidence of a distant spread; the remainder had proven metastases in the skeleton (30), lung (14), liver (10) or in multiple systems (11).

TABLE I.—*Positive receptor assay according to menstrual status and site of biopsy. The difference in the Guy's series between pre- and postmenstrual groups is significant* ($\chi^2=6.25$, $P<0{\cdot}01$). *For this reason only postmenopausal patients were analysed for site. See text for definition of positive* (+) *and equivocal* (±) *assay*

| | Guy's | Assay | | | Edinburgh | Assay | | |
|---|---|---|---|---|---|---|---|---|
| | Total | + | ± | − | Total | + | ± | − |
| Premenopausal | 15 | 5 | 0 | 10 | 11 | 4 | 2 | 5 |
| Postmenopausal | 57 | 37 | 4 | 16 | 36 | 27 | 1 | 8 |
| | 72 | 42 | 4 | 26 | 47 | 31 | 3 | 13 |
| *Postmenopausal* | | | | | | | | |
| Breast | 32 | 21 | 2 | 9 | 29 | 20 | 1 | 8 |
| Nodes | 7 | 3 | 0 | 4 | 1 | 1 | 0 | 0 |
| Skin | 18 | 13 | 2 | 3 | 6 | 6 | 0 | 0 |
| | 57 | 37 | 4 | 16 | 36 | 27 | 1 | 8 |

*Histological review*

One hundred and twenty-eight tumours were available for histological assessment, including material from some patients who were omitted from the main analysis for clinical reasons. Of these, 100 were from postmenopausal patients and, when the relationship between histological score and ER measurement was analysed by a non-parametric test for trend (Cox, 1969), it was found that half the tumours in the inadequate and doubtful histological categories (2, 3, 4) were ER-negative, compared with only one fifth of tumours in the other groups (5, 6, 7) (Table II).

TABLE II.—*The proportion of* $ER^-$ *tumours according to histological score in postmenopausal patients from both centres. The sum of the T values for Cox's test for trend from the two series gives a Z score of* 2·06, *significant at the* 5% *level when referred to the normal distribution. Definition of* $ER^-$ *result in text*

| | Histological score | | | | | | | | |
|---|---|---|---|---|---|---|---|---|---|
| | Guy's | | | Edinburgh | | | Combined | | |
| | 2, 3, 4 | 5 | 6, 7 | 2, 3, 4 | 5 | 6, 7 | 2, 3, 4 | 5 | 6, 7 |
| ER− | 6 | 5 | 6 | 5 | 1 | 3 | 11 | 6 | 9 |
| Total | 13 | 21 | 30 | 10 | 12 | 15 | 23 | 33 | 45 |
| % | | | | | | | 48 | 18 | 20 |

*Response to therapy*

Combining the data from both centres, 119 assessable patients had been treated by endocrine therapy. Of these, 26 were premenopausal and were treated by ophorectomy; the rest were postmenopausal and were treated by hypophysectomy or $^{90}$Y implantation of the pituitary, tamoxifen, stilboestrol, androgens (generally fluoxymesterone), prednisone or adrenalectomy. The number of patients in each treatment group, and the clinical response to therapy related to the result of the oestrogen-receptor assay, are shown in Table III. All responses were graded according to the UICC criteria, and agreed upon at external review.

TABLE III.—*The clinical response (CR+PR) according to result of ER assay in 116 patients treated by endocrine therapy. The difference in response rate between ER⁺ (including equivocal) and ER⁻ groups is significant* ($\chi^2$=8·97, P<0·0025). *See text for definitions of clinical response and oestrogen-receptor status*

| | No. treated | $ER^+$ | $ER^\pm$ | $ER^-$ |
|---|---|---|---|---|
| Ophorectomy | 26 | 6/9 | 0/2 | 1/15 |
| Hypophysectomy | 18 | 3/10 | 1/1 | 0/7 |
| Stilboestrol | 24 | 7/19 | 0/0 | 0/5 |
| Tamoxifen | 34 | 6/24 | 1/2 | 2/8 |
| Androgens | 12 | 1/8 | 2/2 | 0/2 |
| Others | 5 | 1/3 | 0/0 | 0/2 |
| Total | 119 | 24/73 | 4/7 | 3/39 |

Of the 80 patients who had measurable ER protein in their tumours, 28 achieved partial or complete remission of their disease (35%). Only 3 (8%) of the 39 patients with undetectable oestrogen receptor responded to therapy. In the group of patients treated by tamoxifen (generally at a dose of 30 mg/day) the response rate was similar, whether ER protein was present or absent from the tumour.

The absolute values of the oestrogen-receptor concentration in the 44 patients treated by endocrine surgery are shown in Fig. 1. Response to ophorectomy was associated with the highest levels of ER protein, but response to hypophysectomy or any of the additive therapies was not (Fig. 2).

FIG. 1.—ER (absolute values) for patients from Guy's and Edinburgh treated by endocrine surgery. ■ complete remissions; ▨ partial remission; ▩ no change; □ progressive disease. The abscissa represents the cut-off point for each assay.

DISCUSSION

The number included in this study represents all eligible patients over a 30-month period, but it is small because in both centres many patients are treated by chemotherapy or combinations of chemotherapy and endocrine therapy. It is noteworthy that 23% of all those referred were withdrawn from the study after external review. This partly reflects organizational problems, but it is worth stressing that in many cases loss was due to inadequate clinical information.

It is of interest that the proportion of ER⁻ tumours was related to the histological score. Although only one patient was excluded because of a histologically in-

FIG. 2.—ER (absolute values) for patients from Guy's and Edinburgh treated by hormones. Key as for Fig. 1.

adequate tumour biopsy specimen, a further 11 patients had a doubtful histological score (3 or 4) and no detectable ER protein. In addition, 3 patients who were excluded from analysis for clinical reasons, also had doubtfully adequate pathology and $ER^-$ tumours. This could mean a total loss to study of 10% (15/156) if more stringent pathological criteria were accepted, which underlines the need for providing adequate tumour material for the biochemist. Previously, Rosen *et al.* (1975) have described low levels of oestrogen-receptor activity in both primary and metastatic tumours with low cellularity.

The overall response to therapy in our study was 27%. Patients with $ER^+$ tumours were 4–5 times more likely (8–9 times in the ophorectomy group) to respond than those with $ER^-$ tumours. In general, patients with $ER^-$ tumours had only an 8% chance of responding to endocrine therapy, compared with 33% in $ER^+$ tumours. This may not be true for tamoxifen; in our study, although the numbers were small, the response rates were similar whether the receptor protein was present or absent. In the overview by McGuire *et al.* (1975), 47 patients had been treated with anti-oestrogens, mainly nafoxidine, with an overall response of 29%, and 18% in those patients whose tumours were $ER^-$. The relationship of response to tamoxifen therapy and oestrogen-receptor status is therefore not so certain, and more data should be sought to determine whether it is influenced by age, site of disease or other parameters.

Our results are somewhat less encouraging than those previously reported, but provide further evidence that the oestrogen-receptor assay is a prognostic aid in selecting certain forms of endocrine therapy for patients with advanced breast cancer. Nevertheless, it may not be justifiable to perform an open biopsy for receptor assays in a patient being treated by additive hormones, as the morbidity from this procedure may be greater than that from the therapy. On the other hand, it is probably wrong to contemplate ablative endocrine surgery for a patient whose tumour does not contain ER protein. With increasing use of non-endocrine methods of treatment, it is now unlikely that more data will be acquired on this point.

In this study, we have tried to highlight some of the organizational problems involved if oestrogen-receptor assays were to be offered as a service to all patients with breast cancer. Whether such a service is justifiable at present is in doubt, and may have to await improvement in the degree of prediction, possibly by the inclusion of assays of other receptor proteins.

We are very grateful to the Cancer Research Campaign, Imperial Cancer Research Fund, and Tenovus for financial help during the period of the study. We would like to thank other members of the British Breast Group, particularly Professor Keith Griffiths and Dr R. D. Bulbrook for their help and suggestions; also Mr Michael Baum for some of the Guy's external reviews. Our thanks are also due to other members of the Edinburgh Combined Breast Clinic (Dr A. O. Langlands, Dr Helen J. Stewart, Mr T. Hamilton and Dr I. J. McFadyen) who treated many of the Edinburgh patients included in this study. We thank Mrs Gillian Raab of the Medical Computing and Statistics Unit, University of Edinburgh, who performed the statistical analysis for Table III.

### REFERENCES

BRITISH BREAST GROUP (1974) Assessment of response to treatment in advanced breast cancer. *Lancet*, ii, 38.

COX, D. R. (1969) *Analysis of Binary Data*, London: Methuen, p. 61.

HAWKINS, R. A., HILL, A. & FREEDMAN, B. (1975) A simple method for the determination of oestrogen receptor concentrations in breast tumours and other tissues. *Clin. Chim. Acta*, **64**, 203.

HAYWARD, J. L., RUBENS, R. D., CARBONE, P. P., HEUSON, J. C., KUMAOKA, S. & SEGALOFF, A. (1977) Assessment of response to therapy in advanced breast cancer. *Br. J. Cancer*, **35**, 292.

KING, R. J. B., HAYWARD, J. L., KUMAOKA, S. & YAMAMOTO, H. (1977) Comparison of soluble oestrogen and progestin receptor content of primary breast tumours from Japan and Britain. *Eur. J. Cancer*, **13**, 967.

KING, R. J. B., BARNES, D. M., HAWKINS, R. A., LEAKE, R. E., MAYNARD, P. V. & ROBERTS, M. M. (1978) Measurement of oestradiol receptors by five institutions on common tissue samples. *Br. J. Cancer*, **38**, 428.

MCGUIRE, W. L., CARBONE, P. P., SEARS, M. E. & ESCHER, G. C. (1975) In *Oestrogen Receptors in Human Breast Cancer*, ed. W. L. McGuire, P. P. Carbone & E. P. Vollmer. New York: Raven Press.

ROSEN, P. P., MENENDEZ-BOTET, C. J., NISSELBAUM, J. S., URBAN, J. A., MIKE, V., FRACCHIA, A. & SCHWARTZ, M. K. (1975) Pathological review of breast lesions analyzed for estrogen receptor protein. *Cancer Res.*, **35**, 3187.

SCATCHARD, G. (1949) The attraction of proteins for small molecules and ions. *Ann. N.Y. Acad. Sci.*, **51**, 660.